# Antithrombotic management of patients with atrial fibrillation—Dutch anticoagulant initiatives anno 2020

**DOI:** 10.1007/s12471-020-01446-6

**Published:** 2020-08-11

**Authors:** G. Chu, J. Seelig, E. M. Trinks-Roerdink, G. J. Geersing, F. H. Rutten, J. R. de Groot, M. V. Huisman, M. E. W. Hemels

**Affiliations:** 1 Department of Thrombosis and Haemostasis, Leiden University Medical Centre, Leiden, The Netherlands; 2 Department of Cardiology, Rijnstate, Arnhem, The Netherlands; 3 Department of Internal Medicine, Cardiovascular Research Institute Maastricht, Maastricht, The Netherlands; 4 Department of General Practice, Julius Centre for Health Sciences and Primary Care, University Medical Centre Utrecht, Utrecht University, Utrecht, The Netherlands; 5 Department of Cardiology, Heart Center, Amsterdam University Medical Center, University of Amsterdam, Amsterdam, The Netherlands; 6 Department of Cardiology, Radboud University Medical Centre, Nijmegen, The Netherlands

**Keywords:** Atrial fibrillation, Anticoagulation, Registry, Adherence, Stroke and bleeding risk, Integrated care

## Abstract

In recent years, as more and more experience has been gained with prescribing direct oral anticoagulants (DOACs), new research initiatives have emerged in the Netherlands to improve the safety and appropriateness of DOAC treatment for stroke prevention in patients with atrial fibrillation (AF). These initiatives address several contemporary unresolved issues, such as inappropriate dosing, non-adherence and the long-term management of DOAC treatment. Dutch initiatives have also contributed to the development and improvement of risk prediction models. Although fewer bleeding complications (notably intracranial bleeding) are in general seen with DOACs in comparison with vitamin K antagonists, to successfully identify patients with high bleeding risk and to tailor anticoagulant treatment accordingly to mitigate this increased bleeding risk, is one of the research aims of recent and future years. This review highlights contributions from the Netherlands that aim to address these unresolved issues regarding the anticoagulant management in AF in daily practice, and provides a narrative overview of contemporary stroke and bleeding risk assessment strategies.

## Dutch contribution to the field

- Investigating the impact of adherence, persistence and incorrect dosing of DOACs in AF patients.
- Determining the safety of switching anticoagulants (from VKA to DOAC) in frail, elderly AF patients.
- Evaluating whether integrated care for AF can be safely organised in primary care.
- Tailoring DOAC intensity to the individual patient based on dose reduction criteria alone.

## Introduction

Substantial improvements have recently been made in the anticoagulant management of stroke prevention in atrial fibrillation (AF) [1]. These improvements are continuously needed as AF and AF-related complications such as stroke not only affect an ever-increasing number of patients worldwide, but also impose a great burden on healthcare expenditure [2, 3].

The introduction of direct oral anticoagulants (DOACs) for stroke prevention in AF was one of the major improvements in recent years. Although DOACs are increasingly being accepted and have recently replaced vitamin K antagonists (VKAs) as the preferred anticoagulant among new patients with AF [4–6], several key concerns with respect to patient management and the organisation of anticoagulant therapy care remain. For example, (i) non-adherence and non-persistence to DOACs, (ii) safety and efficacy of DOACs in specific patient groups (such as the frail elderly), (iii) adequate monitoring for comorbidities (e.g. heart failure), and (iv) the issue of who is responsible for the long-term anticoagulant control, are all remaining knowledge gaps in the antithrombotic management in patients with AF.

Another major effort to improve anticoagulant management in AF patients was the development of stroke and bleeding risk prediction models, which underline differences in stroke and bleeding risks between individual patients. In the past decade, several scores have been developed and validated, all with the aim to optimise the safety and appropriate use of anticoagulation therapy in AF [7–13]. However, despite all efforts and albeit suboptimal for prediction of stroke, only the CHA_2_DS_2_-VASc score is currently adopted in international AF guidelines [14–16]. Risk assessment strategies that implement *both* stroke and bleeding risk scores, on which a tailored individualised anticoagulant treatment plan could be based, such as with the ABC (Age, Biomarker, Clinical history) score, have been proposed but not implemented in international AF guidelines [17].

In this review, we highlight ongoing Dutch initiatives that address unresolved issues regarding anticoagulant management in AF in daily practice and we provide a narrative overview of contemporary stroke and bleeding risk assessment strategies.

## Dutch initiatives

### DUTCH-AF registry—inappropriate dosing, non-adherence and non-persistence to anticoagulants

In contrast to VKAs, DOACs do not require routine anticoagulation monitoring. As a result, concerns were raised about patient adherence and persistence to DOACs [18]. In other common chronic diseases, non-adherence occurs frequently and this obviously affects the course of disease negatively [19, 20]. In line with these findings, recent practice-based and insurance claims data have provided evidence that non-adherence and non-persistence to anticoagulant therapy occur in AF patients as well, with similar adverse effects on safety and efficacy outcomes [21–27].

Another issue related to prescribing DOACs is inappropriate dosing. Several retrospective studies and prospective registries have shown that inappropriate dosing (most often underdosing) of DOACs occurs frequently in 7 to 40% of AF patients [28–30]. This may possibly be associated with an increased risk for major adverse cardiac or bleeding events [28].

The aims of the ongoing DUTCH-AF registry (ZonMw project numbers 848050006 and 848050007) are to assess the effects of inappropriate dosing, non-adherence and non-persistence on AF-related clinical outcomes, such as stroke and bleeding, and to identify predictors of inappropriate dosing, non-adherence and non-persistence. Therefore, data on the anticoagulant management and clinical course of Dutch patients with newly diagnosed AF are being collected. This nationwide registry intends to enrol 6000 patients with a follow-up of at least 2 years. Data on patient demographics, relevant medical history, pattern and anticoagulant treatment of AF, and pharmacy medication dispensing data will be collected. Also, detailed information is being collected on AF-related adverse events such as bleeding, ischaemic events and AF-related hospitalisation. As of January 2020, over 3000 patients have been included.

A unique feature of DUTCH-AF is that a prospective study on dosing, non-adherence and non-persistence to anticoagulation therapy will be conducted simultaneously. A composite questionnaire regarding anticoagulation adherence and beliefs about drugs will be sent to a subset of patients to identify predictors of non-adherence. Finally, with this prospective study, DUTCH-AF aims to validate and refine currently applied bleeding risk assessment models, and to integrate stroke and bleeding assessment models.

### FRAIL-AF—optimal anticoagulant in frail elderly AF patients

Based on the landmark DOAC randomised controlled trials (RCTs), current international guidelines recommend DOACs as the first choice of anticoagulation therapy in AF [14, 15, 31–34]. However, the positive findings on the efficacy and safety of DOACs may not be generalisable to frail elderly AF patients, as they were under-represented in these RCTs. Whilst being distinctly different than the general population, these patients often encounter difficulties with anticoagulant management and have a high risk for bleeding, stroke and unplanned hospitalisations [35, 36]. Factors such as multimorbidity, polypharmacy, insufficient vitamin K intake and an altered body composition (relatively less muscle mass and more fatty tissue) could contribute to the challenges in anticoagulant management in frail elderly AF patients [37]. Moreover, cognitive and social factors such as social isolation, mood disorders and cognitive decline, which are not accounted for in most studies, could also hamper anticoagulant treatment (e.g. due to non-persistence which—as explained above—may be more likely in patients treated with a DOAC).

Currently, there are insufficient data on the association between AF, frailty, older age, anticoagulant management and clinical outcomes to guide optimal anticoagulant management in this specific population. In particular, it is unknown whether switching to a DOAC in frail elderly AF patients, treated with VKA, would result in fewer bleeding complications, as the clinical practice data that are available on the safety and effectivity of switching anticoagulant treatment are confounded by the reason to switch [38].

The Dutch FRAIL-AF RCT aimed at providing insights into the optimal anticoagulant treatment strategy in frail elderly AF patients [39]. This multicentre pragmatic open label registry-based clinical trial was designed to assess whether switching from VKA to DOAC reduces the risk of major and clinically relevant non-major bleeding in frail elderly with AF (primary endpoint). The FRAIL-AF study will include frail elderly patients (≥75 years with a Groningen Frailty Indicator ≥3) with non-valvular AF [40]. Patients randomised to the intervention group will switch from VKA to DOAC-based management. The control group continues with their VKA treatment.

### ALL-IN—Integrated AF care in primary care

AF is closely intertwined with other common chronic diseases such as type 2 diabetes, chronic lung diseases, hypertension and heart failure. Integrated care, in which AF and its comorbidities are managed in a multidisciplinary setting, is suggested as a possible strategy for promoting guideline adherence and improving patient outcomes [14]. Such integrated AF care in secondary care has been shown to be effective in reducing all-cause mortality, cardiovascular hospitalisation and healthcare costs [41–44].

The ALL-IN study, a cluster randomised trial, was designed to assess safety, efficacy and cost-effectiveness of integrated AF care in primary practice [45]. AF patients were enrolled from primary care. The primary endpoint was all-cause mortality during a follow-up duration of 2 years. Practices randomised to the intervention arm provided integral AF care, consisting of regular check-ups and—in case of VKA use—INR measurements performed by the practice nurse, and easy access to consultation with cardiologists and experts in anticoagulation. Practices randomised to the control group provided care as usual.

A 45% reduction in all-cause mortality was observed in the intervention group compared with the control group (HR 0.55, 95% CI 0.37 to 0.82) [46]. Bleeding and stroke rates were similar between the intervention and control group, whereas the study did observe that the reduction in all-cause mortality was greater for non-cardiac causes of death as compared with cardiovascular death. As such, the effect on mortality was not explained by better anticoagulation monitoring. However, it was speculated that management in primary care for patients eligible for referral back to their general practitioner may allow more attention to be given to the comorbidities of AF patients, since general practice typically incorporates a holistic approach. Furthermore, integrated AF care in primary care could offer a solution for managing the increasing prevalence of AF patients (i.e. the AF epidemic) and maintaining the accessibility of VKA monitoring when anticoagulation clinics are expected to close down as more and more patients are treated with DOACs.

## Contemporary stroke and bleeding risk assessment strategies

A multitude of risk prediction models have been designed to objectively assess and weigh the different stroke and bleeding risks of individual patients before initiating anticoagulant treatment. The CHA_2_DS_2_-VASc score, currently the only endorsed score in international guidelines, is used to identify AF patients at low risk for stroke, in whom anticoagulant treatment can be safely withheld [14, 15]. Although several modifications to the CHA_2_DS_2_-VASc score have resulted in an improved predictive performance [47–51], these modifications have not been adopted by international AF guidelines.

For bleeding risk assessment, the HAS-BLED score was briefly recommended in guidelines over the HEMORR_2_HAGES and the ATRIA score to identify AF patients with an increased bleeding risk for close monitoring [8, 13, 14, 52]. However, due to its moderate discriminatory abilities for major bleeding, especially in the elderly in which bleeding prediction matters most [8, 53] and its inability to guide anticoagulant treatment [9, 54], later or subsequent editions of the international guidelines have dropped the recommendation for a specific bleeding risk score. Instead, a list of modifiable and non-modifiable bleeding risk factors was included which clinicians could target to modify bleeding risk. Anticoagulant treatment should not be withheld based on bleeding risk assessment scores [14, 55].

In recent years, the quest for developing and improving risk prediction scores continued, all with the aim to either improve the risk prediction capabilities, to fit into the modern era of DOACs and/or to tailor anticoagulant treatment to the individual patient. Examples are (in chronological order): the ORBIT bleeding score, ABC score, GARFIELD-AF risk tool, Anticoagulation-specific Bleeding Score (ABS), and a model derived from the RE-LY trial [10, 12, 17, 56, 57]. Three interesting developments are seen in these newly developed risk models: (i) the development of an integrated risk assessment tool that allows for simultaneous calculation of stroke, bleeding and mortality risk, (ii) the addition of biomarkers and ECG markers to improve the predictive capabilities of these risk scores [51, 58], and (iii) the possibility to tailor anticoagulant treatment to the individual patient, based on the calculated stroke and bleeding risk.

Both the GARFIELD-AF risk tools [59] and the ABC scores [10, 17, 60] are continuous models which provide the opportunity to simultaneously estimate stroke, bleeding and mortality risk. Interestingly, the ABC score incorporates biomarkers to assess these risks. It was shown that high-sensitivity troponin T (hsTnT) and N‑terminal B‑type natriuretic peptide (NT-proBNP) were independently associated with stroke and systemic embolism, and hsTnT and growth differentiation factor-15 (GDF-15) with major bleeding. One important strength in the development of the ABC score is the analysis of treatment interaction for determining the optimal anticoagulant per risk profile, based on a net clinical outcome analysis.

The most recently published model, constructed by a Dutch working group using data from the RE-LY trial, provides guidance on anticoagulant management in AF patients as well [57]. This model estimated the absolute treatment effect of warfarin, dabigatran 150 mg and dabigatran 110 mg, based on the 5‑year ischaemic stroke/systemic embolism and bleeding risk. This calculator could help make an informed decision on the optimal dabigatran dosage, based on the weight assigned to stroke and bleeding risk.

## Future developments of Dutch initiatives

Integrating data from electronic medical records (EMR) into a clinical registry such as DUTCH-AF would ensure the continuity of the registry and allow for long-term follow-up at lower study costs and administrative burden. In collaboration with the Netherlands Heart Registry and EMR vendors, the dataset required for DUTCH-AF will be implemented in EMR systems in the near future. Moreover, as the Netherlands Heart Registry centralises all data from various national cardiovascular registries (e.g. cardiac device registry and percutaneous coronary intervention registry), cross-talk between registries will be possible. Data from one registry could be applicable to all the other registries a patient is enrolled in, without the need for additional follow-up separately.

Connecting these registries would enable the opportunity for future registry-based randomised controlled trials (R-RCT) or trials within cohort designs. Multiple R‑RCTs have already been performed in the SWEDEHEART, Sweden’s online cardiac registry [61]. Benefits seen with R‑RCTs were swifter enrolments, reduction of costs and loss to follow-up compared with standard site-based follow-up. It was shown that the costs of the TASTE trial were approximately US$ 350,000, or $50 per patient, which is considerably lower than other clinical trials on average [62].

For improving risk assessment models, the implementation of risk scores in EMR systems could be valuable as well. The integration of such models in EMR systems would enable synchronisation with the bulk of data clinicians continuously collect from patients for automated real-time risk calculation. We hypothesise this could provide a novel means to improve these risk models.

Implementing risk scores in EMR systems would also facilitate viewing risk as a dynamic process, as score items such as age will and kidney function may often change over time. Recent studies have shown that changes in CHA_2_DS_2_-VASc and HAS-BLED scores were of more importance in predicting ischaemic stroke and bleeding, respectively, than the scores as assessed at baseline [63–65]. Finally, implementation of risk assessment tools in EMR systems can also be used as a quality of care parameter.

## Conclusion

Patients and their physicians continuously encounter challenges with the appropriate use and complications of DOAC treatment in daily practice. The creation of a national AF registry provides the possibility to evaluate the appropriateness and disparities of contemporary anticoagulant care. Embedding anticoagulant control in integrated AF care, which requires a holistic approach and close collaboration between general practitioners and specialists, could partially address these daily encountered challenges effectively and promptly. The results of the studies highlighted in this review will provide important information for clinicians and patients for informed decision-making regarding the provided anticoagulant care.

## References

[1] Freedman B (2018). Major progress in anticoagulant uptake for atrial fibrillation at last: does it translate into stroke prevention?. Eur Heart J.

[2] Chugh SS, Havmoeller R, Narayanan K (2014). Worldwide epidemiology of atrial fibrillation: a Global Burden of Disease 2010 Study. Circulation.

[3] Stewart S, Murphy NF, Walker A, McGuire A, McMurray JJ (2004). Cost of an emerging epidemic: an economic analysis of atrial fibrillation in the UK. Heart.

[4] van den Heuvel JM, Hövels AM, Büller HR, Mantel-Teeuwisse AK, de Boer A (2018). Maitland-van der Zee AH. NOACs replace VKA as preferred oral anticoagulant among new patients: a drug utilization study in 560 pharmacies in The Netherlands. Thromb J.

[5] Marzec LN, Wang J, Shah ND (2017). Influence of Direct Oral Anticoagulants on Rates of Oral Anticoagulation for Atrial Fibrillation. J Am Coll Cardiol.

[6] Camm AJ, Accetta G, Ambrosio G (2017). Evolving antithrombotic treatment patterns for patients with newly diagnosed atrial fibrillation. Heart.

[7] Lip GYH, Nieuwlaat R, Pisters R, Lane DA, Crijns HJGM (2010). Refining Clinical Risk Stratification for Predicting Stroke and Thromboembolism in Atrial Fibrillation Using a Novel Risk Factor-Based Approach: the euro heart survey on atrial fibrillation. Chest.

[8] Pisters R, Lane DA, Nieuwlaat R, de Vos CB, Crijns HJ, Lip GY (2010). A novel user-friendly score (HAS-BLED) to assess 1-year risk of major bleeding in patients with atrial fibrillation: the Euro Heart Survey. Chest.

[9] Olesen JB, Lip GY, Lindhardsen J (2011). Risks of thromboembolism and bleeding with thromboprophylaxis in patients with atrial fibrillation: A net clinical benefit analysis using a ’real world’ nationwide cohort study. Thromb Haemost.

[10] Hijazi Z, Oldgren J, Lindbäck J (2016). The novel biomarker-based ABC (age, biomarkers, clinical history)-bleeding risk score for patients with atrial fibrillation: a derivation and validation study. Lancet.

[11] Fox KAA, Lucas JE, Pieper KS (2017). Improved risk stratification of patients with atrial fibrillation: an integrated GARFIELD-AF tool for the prediction of mortality, stroke and bleed in patients with and without anticoagulation. Bmj Open.

[12] O’Brien EC, Simon DN, Thomas LE (2015). The ORBIT bleeding score: a simple bedside score to assess bleeding risk in atrial fibrillation. Eur Heart J.

[13] Fang MC, Go AS, Chang Y (2011). A New Risk Scheme to Predict Warfarin-Associated Hemorrhage. The ATRIA (Anticoagulation and Risk Factors in Atrial Fibrillation) Study. J Am Coll Cardiol.

[14] Kirchhof P, Benussi S, Kotecha D (2016). ESC guidelines for the management of atrial fibrillation developed in collaboration with EACTS. Eur Heart J.

[15] January CT, Wann LS, Calkins H (2019). 2019 AHA/ACC/HRS Focused Update of the 2014 AHA/ACC/HRS Guideline for the Management of Patients With Atrial Fibrillation: A Report of the American College of Cardiology/American Heart Association Task Force on Clinical Practice Guidelines and the Heart Rhythm Society in Collaboration With the Society of Thoracic Surgeons. Circulation.

[16] van Doorn S, Debray TPA, Kaasenbrood F (2017). Predictive performance of the CHA2DS2-VASc rule in atrial fibrillation: a systematic review and meta-analysis. J Thromb Haemost.

[17] Berg DD, Ruff CT, Jarolim P (2019). Performance of the ABC Scores for Assessing the Risk of Stroke or Systemic Embolism and Bleeding in Patients With Atrial Fibrillation in ENGAGE AF-TIMI 48. Circulation.

[18] Health Council of the Netherlands. New anticoagulants: a well-dosed introduction. The Hague: Health Council of the Netherlands Gezondheidsraad. GR. 2012;07E:2012.

[19] Lemstra M, Nwankwo C, Bird Y, Moraros J (2018). Primary nonadherence to chronic disease medications: a meta-analysis. Patient Prefer Adherence.

[20] de Jager RL, van Maarseveen EM, Bots ML, Blankestijn PJ, SYMPATHY investigators. Medication adherence in patients with apparent resistant hypertension (2018). findings from the SYMPATHY trial. Br J Clin Pharmacol.

[21] Jackevicius CA, Tsadok MA, Essebag V (2017). Early non-persistence with dabigatran and rivaroxaban in patients with atrial fibrillation. Heart.

[22] Borne RT, O’Donnell C, Turakhia MP (2017). Adherence and outcomes to direct oral anticoagulants among patients with atrial fibrillation: findings from the veterans health administration. BMC Cardiovasc Disord.

[23] Collings SL, Lefèvre C, Johnson ME (2017). Oral anticoagulant persistence in patients with non-valvular atrial fibrillation: A cohort study using primary care data in Germany. PLoS ONE.

[24] Forslund T, Wettermark B, Hjemdahl P (2016). Comparison of treatment persistence with different oral anticoagulants in patients with atrial fibrillation. Eur J Clin Pharmacol.

[25] Yao X, Abraham NS, Alexander GC (2016). Effect of Adherence to Oral Anticoagulants on Risk of Stroke and Major Bleeding Among Patients With Atrial Fibrillation. J Am Heart Assoc.

[26] Shore S, Carey EP, Turakhia MP (2014). Adherence to dabigatran therapy and longitudinal patient outcomes: insights from the veterans health administration. Am Heart J.

[27] Kim D, Yang PS, Jang E (2020). The optimal drug adherence to maximize the efficacy and safety of non-vitamin K antagonist oral anticoagulant in real-world atrial fibrillation patients. Europace.

[28] Montrasio G, Coslovsky M, Wiencierz A (2019). Prevalence and risk of DOACs inappropriate dosing in atrial fibrillation. An analysis of the Swiss-AF and BEAT-AF registries. Eur Heart J.

[29] Hirsh Raccah B, Rottenstreich A, Zacks N (2019). Appropriateness of direct oral anticoagulant dosing and its relation to drug levels in atrial fibrillation patients. J Thromb Thrombolysis.

[30] Leef GC, Perino AC, Askari M (2019). Appropriateness of Direct Oral Anticoagulant Dosing in Patients With Atrial Fibrillation: Insights From the Veterans Health Administration. J Pharm Pract.

[31] Connolly SJ, Ezekowitz MD, Yusuf S (2009). Dabigatran versus warfarin in patients with atrial fibrillation. N Engl J Med.

[32] Granger CB, Alexander JH, McMurray JJV (2011). Apixaban versus Warfarin in Patients with Atrial Fibrillation. N Engl J Med.

[33] Patel MR, Mahaffey KW, Garg J (2011). Rivaroxaban versus Warfarin in Nonvalvular Atrial Fibrillation. N Engl J Med.

[34] Giugliano RP, Ruff CT, Braunwald E (2013). Edoxaban versus Warfarin in Patients with Atrial Fibrillation. N Engl J Med.

[35] Wilkinson C, Todd O, Clegg A, Gale CP, Hall M (2018). Management of atrial fibrillation for older people with frailty: a systematic review and meta-analysis. Age Ageing.

[36] Gugganig R, Aeschbacher S, Leong DP (2020). Frailty to predict unplanned hospitalization, stroke, bleeding and death in atrial fibrillation. Eur Heart J Qual Care Clin Outcomes.

[37] Torn M, Bollen WLEM, van der Meer FJM, van der Wall EE, Rosendaal FR (2005). Risks of Oral Anticoagulant Therapy With Increasing Age. Arch Intern Med.

[38] Hellfritzsch M, Adelborg K, Damkier P, et al. Effectiveness and safety of direct oral anticoagulants in atrial fibrillation patients switched from vitamin K antagonists: A systematic review and meta-analysis. Basic Clin Pharmacol Toxicol. 2019. 10.1111/bcpt.13283. [Epub ahead of print] 10.1111/bcpt.13283 PMC6973083 31240841

[39] Joosten LPT, van Doorn S, Hoes AW (2019). Safety of switching from vitamin K antagonist to non-vitamin K antagonist oral anticoagulant in frail elderly with atrial fibrillation: rationale and design of the FRAIL-AF randomised controlled trial. Bmj Open.

[40] Steverink N, Slaets J, Schuurmans H, Lis M (2001). Measuring frailty: Developing and testing the GFI (Groningen Frailty Indicator). Gerontologist.

[41] Proietti M, Romiti GF, Olshansky B, Lane DA, Lip GYH (2018). Improved Outcomes by Integrated Care of Anticoagulated Patients with Atrial Fibrillation Using the Simple ABC (Atrial Fibrillation Better Care) Pathway. Am J Med.

[42] Gallagher C, Elliott AD, Wong CX (2017). Integrated care in atrial fibrillation: a systematic review and meta-analysis. Heart.

[43] Pastori D, Farcomeni A, Pignatelli P, Violi F, Lip GYABC (2019). (Atrial fibrillation Better Care) Pathway and Healthcare Costs in Atrial Fibrillation: The ATHERO-AF Study. Am J Med.

[44] Hendriks JML, de Wit R, Crijns HJGM (2012). Nurse-led care vs. usual care for patients with atrial fibrillation: results of a randomized trial of integrated chronic care vs. routine clinical care in ambulatory patients with atrial fibrillation. Eur Heart J.

[45] van den Dries CJ, Oudega R, Elvan A (2017). Integrated management of atrial fibrillation including tailoring of anticoagulation in primary care: study design of the ALL-IN cluster randomised trial. Bmj Open.

[46] van den Dries CJ, van Doorn S, Rutten FH, et al. Integrated management of atrial fibrillation in primary care: results of the ALL-IN cluster randomized trial. Eur Heart J. 2020. 10.1093/eurheartj/ehaa055. [Epub ahead of print] 10.1093/eurheartj/ehaa055 PMC7421774 32112556

[47] Kabra R, Girotra S, Sarrazin VM (2016). Refining Stroke Prediction in Atrial Fibrillation Patients by Addition of African-American Ethnicity to CHA2DS2-VASc Score. J Am Coll Cardiol.

[48] Tomita H, Okumura K, Inoue H (2015). Validation of Risk Scoring System Excluding Female Sex From CHA2DS2-VASc in Japanese Patients With Nonvalvular Atrial Fibrillation—Subanalysis of the J-RHYTHM Registry. Circ J.

[49] Cha M-J, Cho Y, Oh I-Y, Choi E-K, Oh S (2018). Validation of Conventional Thromboembolic Risk Factors in a Korean Atrial Fibrillation Population—Suggestion for a Novel Scoring System, CHA_2_DS_2_-VAK. Circ J.

[50] Chao TF, Lip GY, Liu CJ (2016). Validation of a Modified CHA2DS2-VASc Score for Stroke Risk Stratification in Asian Patients With Atrial Fibrillation: A Nationwide Cohort Study. Stroke.

[51] Alkhouli M, Friedman PA. Ischemic Stroke Risk in Patients With Nonvalvular Atrial Fibrillation: JACC Review Topic of the Week. J Am Coll Cardiol. 2019;74(24):3050–65. 10.1016/j.jacc.2019.10.040 31865973

[52] Gage BF, Yan Y, Milligan PE (2006). Clinical classification schemes for predicting hemorrhage: results from the National Registry of Atrial Fibrillation (NRAF). Am Heart J.

[53] Jaspers Focks J, van Vugt SPG, Albers-Akkers MTH (2016). Low performance of bleeding risk models in the very elderly with atrial fibrillation using vitamin K antagonists. J Thromb Haemost.

[54] Friberg L, Rosenqvist M, Lip GY (2012). Net clinical benefit of warfarin in patients with atrial fibrillation: a report from the Swedish atrial fibrillation cohort study. Circulation.

[55] January CT, Wann LS, Alpert JS (2014). AHA/ACC/HRS Guideline for the Management of Patients With Atrial Fibrillation. A Report of the American College of Cardiology/American Heart Association Task Force on Practice Guidelines and the Heart Rhythm Society. Circulation.

[56] Claxton JNS, MacLehose RF, Lutsey PL (2018). A new model to predict major bleeding in patients with atrial fibrillation using warfarin or direct oral anticoagulants. PLoS ONE.

[57] Stam-Slob MC, Connolly SJ, van der Graaf Y (2019). Individual Treatment Effect Estimation of 2 Doses of Dabigatran on Stroke and Major Bleeding in Atrial Fibrillation. Circulation.

[58] Maheshwari A, Norby FL, Roetker NS (2019). Refining Prediction of Atrial Fibrillation-Related Stroke Using the P_2_-CHA_2_DS_2_-VASc Score. Circulation.

[59] Dalgaard F, Pieper K, Verheugt F (2019). GARFIELD-AF model for prediction of stroke and major bleeding in atrial fibrillation: a Danish nationwide validation study. Bmj Open.

[60] Hijazi Z, Oldgren J, Lindback J (2018). A biomarker-based risk score to predict death in patients with atrial fibrillation: the ABC (age, biomarkers, clinical history) death risk score. Eur Heart J.

[61] Jernberg T, Attebring MF, Hambraeus K (2010). The Swedish Web-system for enhancement and development of evidence-based care in heart disease evaluated according to recommended therapies (SWEDEHEART). Heart.

[62] Wachtell K, Lagerqvist B, Olivecrona GK, James SK, Frobert O. Novel Trial Designs (2016). Lessons Learned from Thrombus Aspiration During ST-Segment Elevation Myocardial Infarction in Scandinavia (TASTE) Trial. Curr Cardiol Rep.

[63] Chang TY, Lip GYH, Chen SA, Chao TF (2019). Importance of Risk Reassessment in Patients With Atrial Fibrillation in Guidelines: Assessing Risk as a Dynamic Process. Can J Cardiol.

[64] Yoon M, Yang PS, Jang E (2018). Dynamic Changes of CHA2DS2-VASc Score and the Risk of Ischaemic Stroke in Asian Patients with Atrial Fibrillation: A Nationwide Cohort Study. Thromb Haemost.

[65] Chao TF, Lip GYH, Lin YJ (2018). Incident Risk Factors and Major Bleeding in Patients with Atrial Fibrillation Treated with Oral Anticoagulants: A Comparison of Baseline, Follow-up and Delta HAS-BLED Scores with an Approach Focused on Modifiable Bleeding Risk Factors. Thromb Haemost.

